# Pneumatic displacement with intravitreal tPA injection versus vitrectomy with sub retinal tPA injection in small and medium sub macular hemorrhages- a multicenter comparative study

**DOI:** 10.1186/s12886-024-03468-9

**Published:** 2024-05-21

**Authors:** Aya Barzelay, Avishai Daniels, Gal Yaakov Cohen, Adiel Barak, Shulamit Schwartz, Gabriel Katz

**Affiliations:** 1 Tel Aviv Sourasky Medical Center, Tel Aviv, 64239 Israel; 2 Sheba Medical Center, Ramat Gan, 5262100 Israel; 3 Sackler Faculty of Medicine, Tel Aviv University, Tel Aviv, 6997801 Israel

**Keywords:** Sub macular hemorrhage, Age related macular degeneration, tPA, Vitrectomy, Intra-vitreal injection

## Abstract

**Purpose:**

Comparing between the visual outcomes and post operative complications of two surgical treatments for sub macular hemorrhage, pars plana vitrectomy with tissue plasminogen activator (tPA) injection procedure, and pneumatic displacement of submacular hemorrhage with intravitreal tPA injection.

**Methods:**

A retrospective chart review of patients with sub macular hemorrhage (SMH) was performed. Data was collected from 150 patients with sub macular hemorrhage. Patients were followed up from the day of admission and up to a year post surgery. Evaluation included visual acuity, optical coherence tomography (OCT), fundus examination and rates of complications.

**Results:**

Pars plana vitrectomy procedure has showed a better visual outcome in small SMH. Comparing complications between the two treatment modalities, no significant difference has been found in the study.

**Conclusions:**

Pars plana vitrectomy and tPA showed a clear advantage with a trend of better visual acuity as well as a significant predictor to better visual acuity for small and medium sub macular hemorrhage.

## Abstract (significance)

**Why carry out this study?:**

• Either Pneumatic Displacement and tPA injection, or Pars Plana Vitrectomy with subretinal tPA injection were shown to be effective in hemo displacement. • There is no consensus among the therapists regarding preferred treatment options for subretinal bleeding.

**What was learned from the study?:**

• PPV + tPA exhibited a clear advantage with a trend of better BCVA at all time points as well as a significant predictor to better BCVA for small and medium SMH. • Comparing complications between the two treatment modalities, we did not find a significant difference. Implying to equal safety to both surgeries.

## Introduction

Sub macular hemorrhage (SMH) is a dreaded complication of choroidal neovascularization, with poor visual prognosis. The hemorrhage lies between the neuroretina and the retinal pigment epithelium (RPE), and by that interferes with diffusion of nutrients and clearance of metabolic waste by RPE. Moreover, the accumulation of blood causes shearing stress to the photoreceptors, and combined with iron toxicity, lead to photoreceptor death [1] and results in poor visual outcome [2, 3].

Treatment options today vary between conservative intra vitreal injection of anti VEGF agents [4], or interventional treatments aiming to mechanically displace the hemorrhage in combination with anti VEGF and/or tissue plasminogen activator (tPA) therapy [5, 6]. Pneumatic displacement (PD) of submacular hemorrhage using expansile gas was first described in 1996, and ever since was proved as an efficient method leading to visual gain [7, 8]. Interestingly, injection of tPA to the vitreous was shown to penetrate the sub retinal space in animal model [9]. Moreover, the addition of intravitreal tPA injection to intravitreal mechanical displacement with gas was shown to improve treatment results by complete displacement of the hemorrhage in 73% of patients, in a large series (*n* = 192) [8]. Another treatment option available is pars plana vitrectomy (PPV) with sub retinal injection of tPA (SRtPA) concluded by air fluid exchange [10, 11]. Each of the methods, either PD and tPA injection or PPV with subretinal tPA injection were shown to be effective in hemo displacement [8, 11]. However, to the best of our knowledge no large randomized clinical trial has been conducted comparing both methods and there is no consensus regarding preferred treatment option.

In this study we compared retrospectively treatment outcomes between either PD and tPA versus PPV with SRtPA concluded with gas air fluid exchange, in patients suffering from SMH.

## Methods

A retrospective chart review of patients with SMH from the years 2/2015 to 5/2020, at Sheba Medical center and at Tel Aviv Medical center was performed. The Institutional Review Board of both medical centers approved the protocol. Data were collected from medical charts of patients who had SMH involving the fovea secondary to exudative AMD.

The baseline evaluation included best corrected visual acuity (BCVA) with a Snellen chart, dilated fundus examination, and spectral domain optical coherence tomography (SD-OCT) by Heildelberg. Baseline characteristics were collected from patients’ charts: age, gender, lens status, glaucoma, prior PPV, coexisting diseases (diabetes, hypertension), anti-coagulation drugs, AMD diagnosis prior to presentation, anti VEGF injections prior to surgery, measured hemorrhage dimensions, central retinal thickness (CRT) on the SD OCT, time to surgery, procedure type (combination of cataract surgery and tamponade type: air, gas or silicone), complications.

The dimensions of SMH were assessed by two measurements: height and area. The height was defined by manual measurements of the distance between the internal limiting membrane (ILM) and the Bruch’s membrane (BM) at the center of the fovea, using a manual ruler on the integrated software of the SD-OCT (Spectralis; Heidelberg, Germany). The area was defined by drawing the circumference of the total hemorrhage on the Heidelberg software on the infra-red fundus picture. The area was then calculated to mm^2 using the Heidelberg software. Patients were divided into three groups based on the size of the SMH: small-sized < 20 mm^2, medium 20–50 mm^2, large > 50 mm^2).

Patients were referred to a treatment modality based on each medical centers’ preference plus a surgeon’s judgement of optimal treatment. In one medical center all patients are referred to PPV + SR tPA, whereas in the second medical center patients are referred to either method based on surgeon’s judgement of optimal treatment, with a tendency to refer large SMH that cross the arcades, to PPV + SR tPA injection and smaller hemorrhages to PD + intravitreal tPA.

Sixty one eyes underwent 23G or 25G vitrectomy, with subretinal injection of 25 micrograms/0.1CC of tPA, concluded with fluid air exchange. Subretinal tPA injection was performed using a 39G tip needle connected to the microdose 1 ml syringe (MedOne, Sarasota, Fl) and to the viscous fluid injection set of the constellation machine. The pressure for the injection was set at 8–10 mm/hg. 46 eyes underwent pneumatic displacement with intravitreal injection of 0.5 mL of 100% SF6, followed by intravitreal injection of 50micrograms/0.1mL of tPA.

The main outcomes were collected at 1, 3 and 12 months after surgery: the displacement of the SMH from the fovea, BCVA and CRT. Secondary outcomes collected were recurrent hemorrhage, cataract surgery, second PPV surgery. Treatment outcomes among the subgroups were compared.

Statistical analysis was performed using SPSS software version 25. Descriptive statistics were performed using means, standard deviations, and ranges for the continuous variables, and frequencies for the discrete variables. Differences between the type of surgeries were assessed using Chi-square tests for the discrete variables, and Mann-Whitney tests for the continuous variables. To assess differences in outcomes (BCVA/CRT) along follow ups between groups, repeated measures ANOVA was conducted. Multivariate analyses for predicting BCVA were assessed using linear regressions that adjusted for significant differences between groups in baseline. P-value lower than 5% is considered to be significant.

## Results

### Study population demographic and baseline characteristics

The differences in the demographic characteristics by procedure type were assessed using Chi-square tests for the discrete variables and Mann-Whitey tests for the continuous variables.

We noted significant differences between groups in several parameters such as phakic status, anti VEGF treatment at baseline, BCVA at baseline as described in Table 1.

**Table 1:** Demographics and baseline characteristics by procedure type

	Vitrectomy	PD	Χ^2^	*P*
**Demographics**				
Gender			0.00	0.96
Male	33 (60%)	25 (59.5%)		
Female	22 (40.0%)	17 (40.5%)		
Age	81.67 ± 7.46	83.33 ± 6.51		0.25
**Baseline characteristics**				
Phakic status			5.89	0.02
Phakic	14 (25.5%)	20 (48.8%)		
Pseudo phakic	41 (74.5%)	21 (51.2%)		
Anti-coagulation drugs	18 (32.7%)	12 (30.0%)	0.08	0.78
Anti VEGF injections before surgery	37 (67.3%)	38 (90.5%)	7.31	< 0.01
AMD diagnosis prior to presentation	42 (76.4%)	40 (95.2%)	6.49	0.01
**Hem dimensions**			15.71	< 0.01
< 20	12 (25.5%)	17 (40.5%)		
20–50	18 (38.3%)	24 (57.1%)		
> 50	17 (36.2%)	1 (2.4%)		
Best corrected visual acuity log mar	1.69 ± 0.58	1.33 ± 0.63		0.01
CRT measured	961.34 ± 379.05	782.07 ± 297.79		0.02
Time from Sx to operation	4.92 ± 4.55	3.79 ± 4.27		0.04

Due to significant pre-surgery differences between the two studied groups, these variables were later controlled by multi-variate models.

As for SMH dimensions, of 89 eyes with measurements of SMH size, 29 eyes (32.5%) had small hemorrhages </= 20 mm^2, 42 eyes (47%) had medium size hemorrhage − 20–50 mm^2, and 18 eyes, 20.2% of eyes had a large hemorrhage =/> 50 mm^2 (Table 1).

### Primary end points- visual acuity, CRT, and complete displacement

Post-surgery parameters were analyzed by SMH dimensions. Due to small sample size in large SMH (over 50mm^2^), subgroup analysis was conducted for small (less than 20mm^2^) and medium sized (20-50mm^2^) hemorrhages only.

### Visual acuity in small and medium sized SMH

**Fig. 1:**
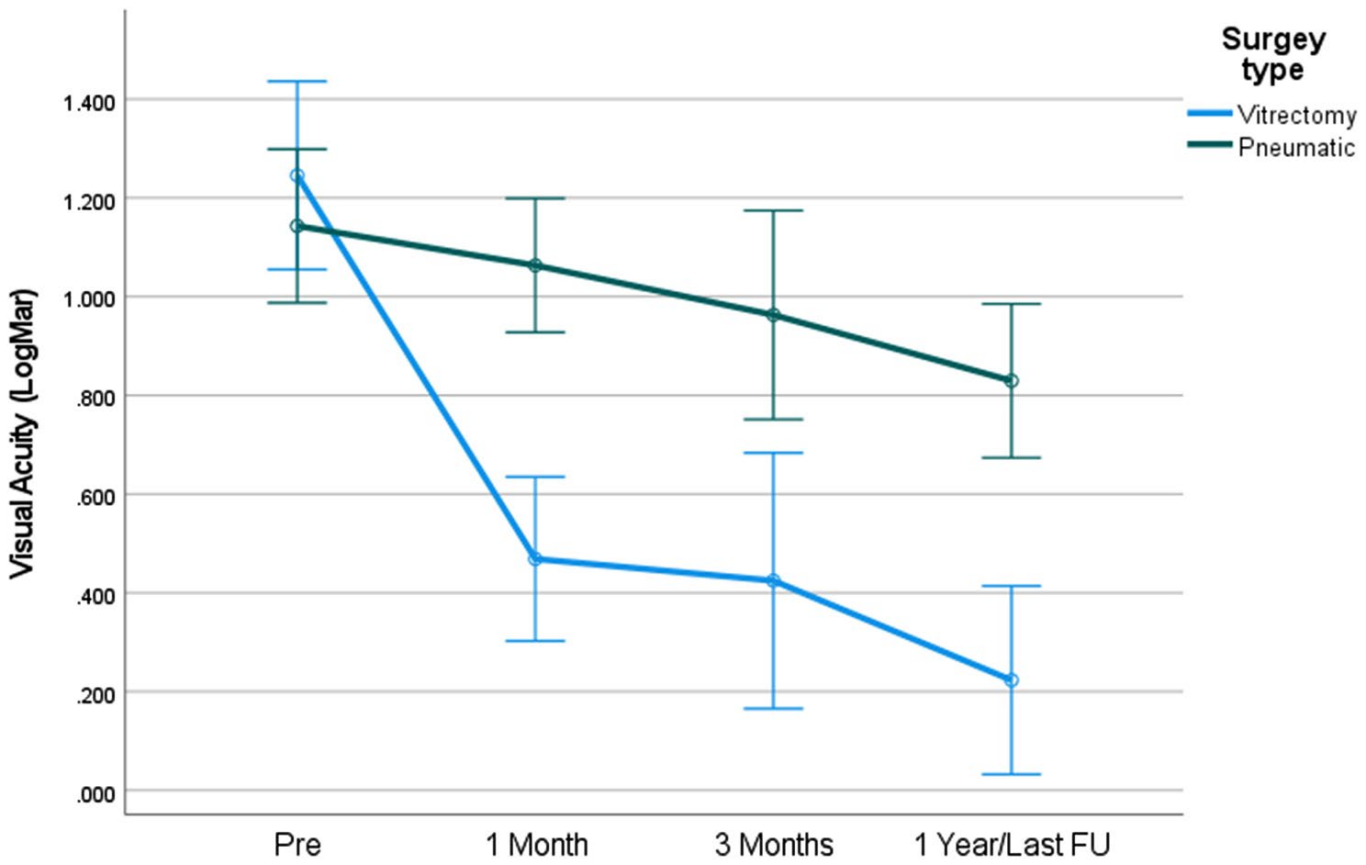
BCVA by surgery type – Hem dimensions < 20

In eyes with small SMH (< 20 mm^2^), a clear trend of better BCVA was noted in the PPV group at all time points (Fig. 1).

**Fig. 2:**
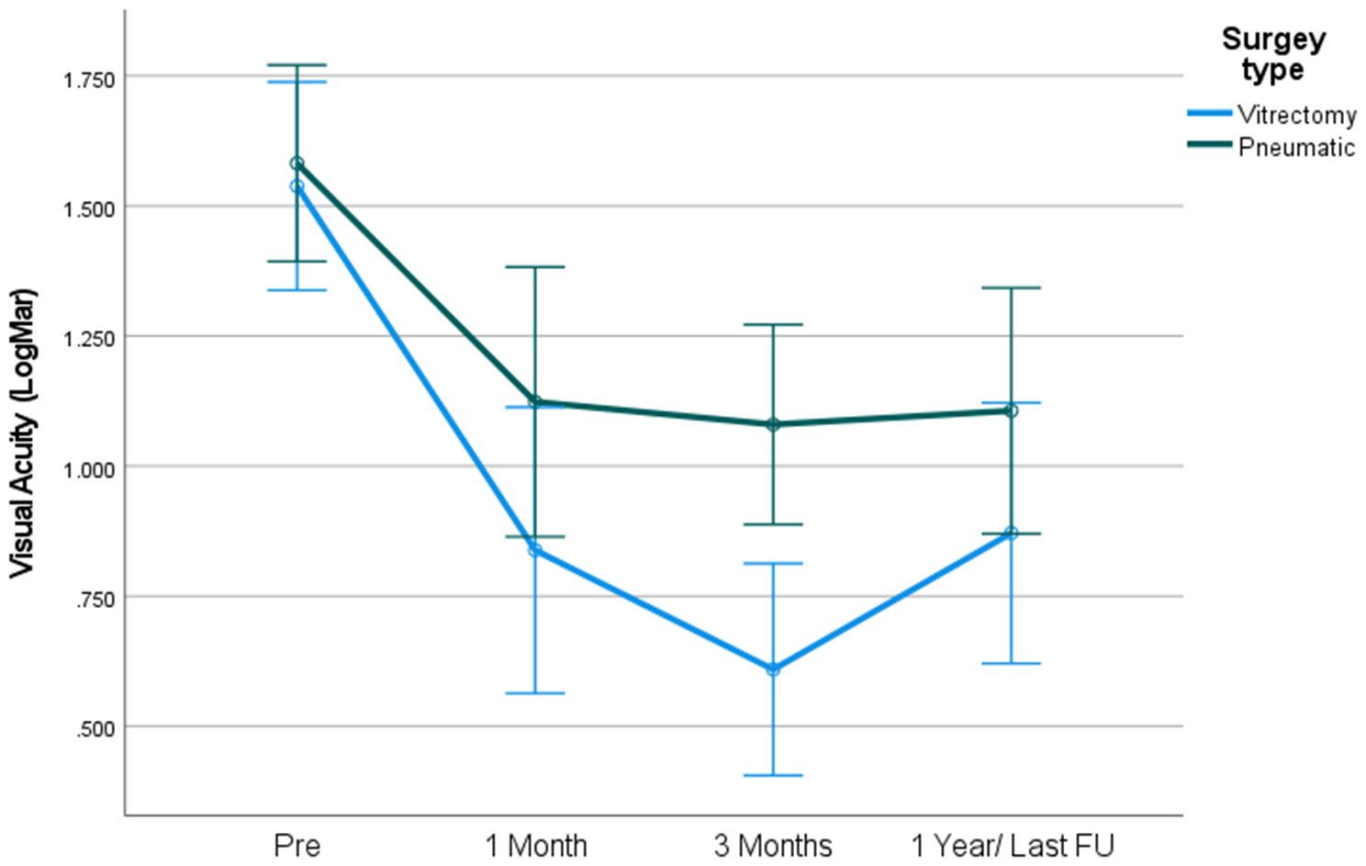
BCVA by surgery type – Hem dimensions 20–50 mm^2

For patients with hemorrhage dimensions of 20–50 mm^2^, a clear trend in BCVA was noted in the PPV group at all time points (Fig. 2).

To note that due to small sample size in each hemorrhage group this difference didn’t achieve statistical significance.

### BCVA gain

In patients with hem dimensions lower than 20mm^2^, PPV surgery achieved higher BCVA gain in comparison with PD (M = 0.77, vs. M= -0.04 *p* < .05 respectively). No difference was found in BCVA gain between surgery type among patients with hem dimensions 20–50 (*p* = .48) .

### Predictors of visual acuity

Multivariate analysis was conducted in order to assess various parameters for predicting visual acuity at different time points after each procedure. The parameters were: Anti coagulation drugs, Anti VEGF injections prior to surgery, baseline visual acuity, hemorrhage dimensions, baseline CRT and time to surgery.

After adjusting for potential confounders including all parameters that were significantly different at baseline among groups (Table 1), we found that patients who underwent PD had lower visual acuity in comparison with PPV at all follow up time points ;1 month (β = 0.279, *p* < .10), 3 months (β = 0.300, *p* < .05), and 1 year/last FU (β = 0.238, *p* < .10). Patients treated with anti-coagulation drugs prior to the procedures, had lower BCVA at 1 year FU (β = 0.232, *p* < .10). SMH larger than 20 mm^2 predicts lower BCVA at 3 months FU (β = 0.257, *p* < .05) compared to SMH smaller than 20 mm^2. Finally, better BCVA at baseline, predicts better BCVA at one (β = 0.353, *p* < .05) and three moths FU (β = 0.282, *p* < .05).

Multivariate analysis was conducted also for predictors of BCVA outcomes for each surgery independent of the other procedure. Predictors for vitrectomy showed an advantage for prior anti VEGF therapy and better BCVA at baseline at one month FU only (β =-0.376, *p* < .1, β = 0.367, *p* < .1 respectively. For PD, prior anti VEGF therapy was found to be a predictor for better BCVA at 12 months (β = 0.388, *p* < .05), as well as better BCVA at baseline (β = 0.515, *p* < .05).

### CRT in small and medium sized SMH

**Fig. 3:**
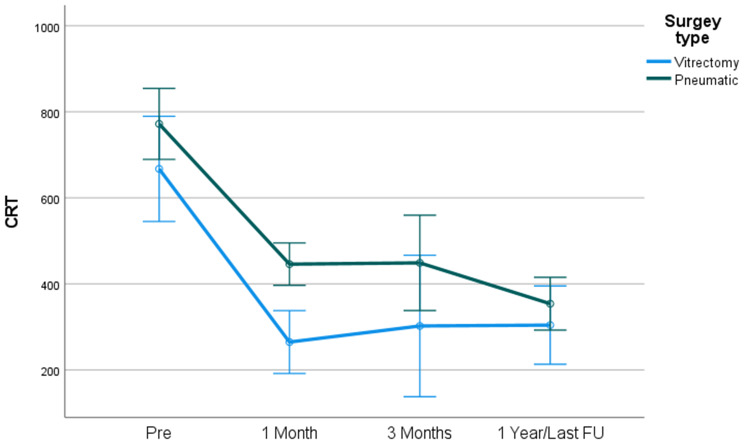
CRT by surgery type – Hem dimensions 20mm^2

In small SMH, a more significant reduction in CRT was found after PPV surgery in comparison with PD at one month and at one year post surgery. (CRT at one-month post-surgery 380.44 ± 293.52 vs. 455.75 ± 148.61, *p* < .05. CRT at 12 months post-surgery 321.67 ± 158 vs. 406.71 ± 253.80 respectively, *p* < .05). (Fig. 3).

**Fig. 4:**
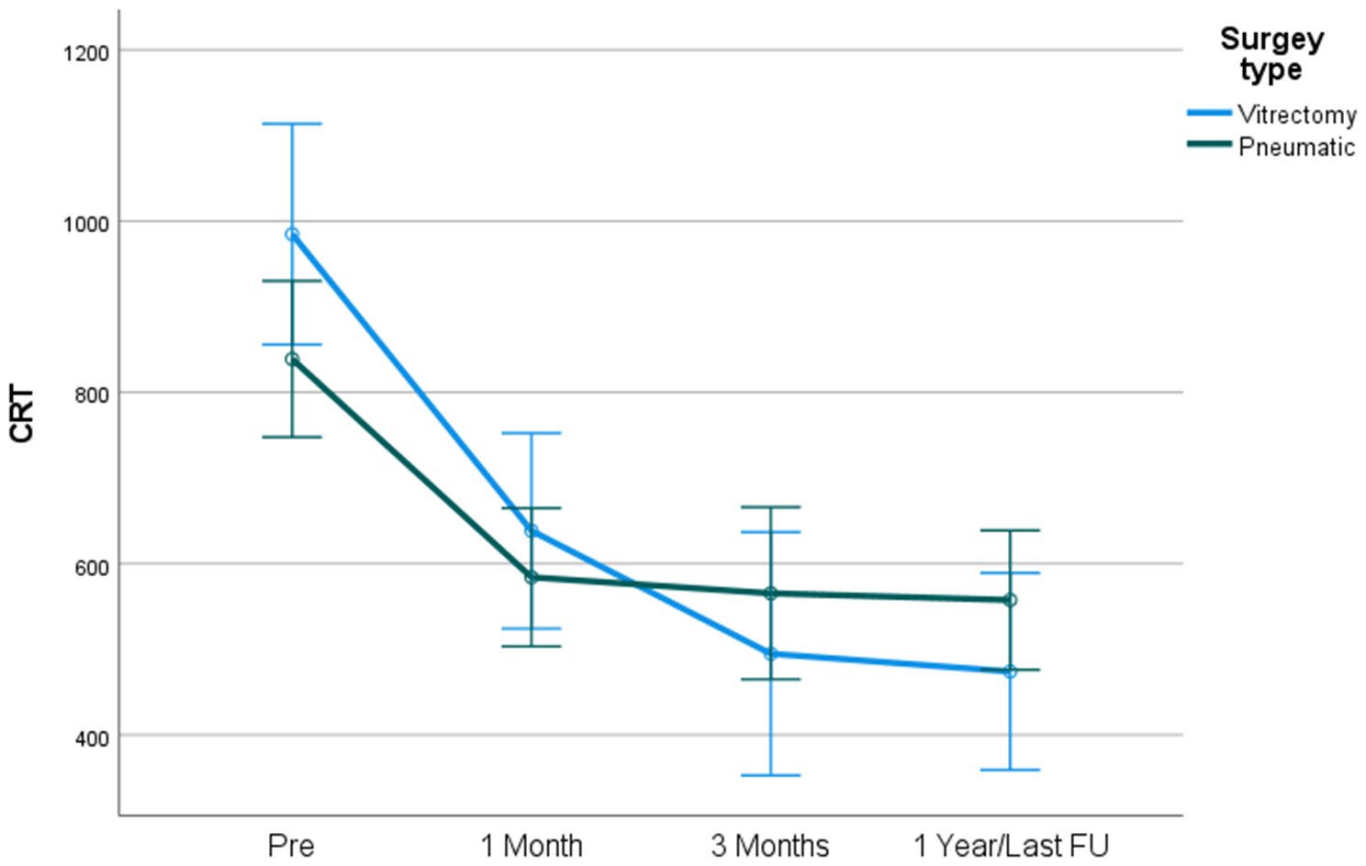
CCRT by surgery type – Hem dimensions 20–50 mm^2

In medium sized SMH, there was no significant difference between groups in CRT (Fig. 4).

### Complete displacement in small and medium sized SMH

In small and medium sized SMH, there was no significant difference between groups in complete displacement of hemorrhage.

## Complications by procedure type

The Differences in the complications by procedure type were assessed using Chi-square tests and Fisher exact tests. As for Phaco surgery within one year, episode of re-bleeding, second surgery or vitreous hemorrhage (VH), there was no significant difference in incidence between groups (Table 2).

**Table 2:** complications by procedure type

	Vitrectomy		PD		Χ^2^	*P*
	*N*	%	*N*	%		
Phaco within 1 Y	5	9.8	4	11.4	0.06	0.99
Re-bleed	6	13.6	7	19.4	0.49	0.48
Redo OP	5	11.6	3	8.3	0.23	0.72
VH	2	4.5	6	16.7	3.23	0.13

## Discussion

In this study we compared real life data of two treatment modalities for SMH. We included a variety of hemorrhage sizes from a large number of patients, from two main medical referral centres. Studied patients were divided into three groups based on hemorrhage size, and analysis was done for major surgery outcomes taking in consideration and adjusting for variation between groups.

### Visual acuity

After adjusting for hemorrhage dimensions, a prominent trend of better BCVA was noted in the PPV group in both SMH that were smaller than 20 mm^2 and between 20 and 500 mm^2. These findings were in accordance with the regression analysis for predictors of BCVA, in which a significant advantage for PPV was found in all time points. Furthermore, and most specifically for BCVA gain, we found a significant advantage for patients with hemorrahges smaller than 20 mm^2 who undergone PPV when compared to PD procedure. Bell et al. compared both treatment procedures for SMH, and didn’t find a significant difference in BCVA between the two treatment modalities [12](Bell, Shulman et al. 2017). To note that in Bells’ study, PD and tPA injection were conducted in a stepwise manner, and as opposed to our study, Bell et al. didn’t perform sub group analysis for different hemorrhages sizes.

There are very few studies that compared PPV + tPA to PD + tPA for SMH. Among which, Jeong et al. compared three treatment modalities for SMH : anti VEGF alone, PD and tPA or PPV + tPA. In their study like in the current study, independent overall improvement in BCVA was noted in all treatment modalities. When analysing per SMH dimensions, Jeong et al. describe an advantage to anti VEGF alone for small SMH, with no significant difference in BCVA between PPV or PD, and advantage in BCVA for PPV + tPA for larger SMH [13]. In our study, we found a trend of better BCVA for PPV + tPA in small and medium SMH. Fassbender et al. reported better BCVA for patients undergoing PPV + tPA, when compared to PD alone [14] however, the lack of tPA injection in the PD group might cause the difference seen when compared to PPV + tPA. Other studies showed the advantage of PPV + tPA to SMH. Treumer et al. [15] didn’t compare to PD, but did show the effectiveness of PPV + tPA with complete displacement and improved BCVA in 87% of patients with large SMH. our report implies to the advantage of PPV + tPA over PD + tPA, with a clear trend of better BCVA for small and medium SMH, as well as significant improved gain in BCVA for small SMH treated with PPV + tPA.

### Predictors for better BCVA

Looking at predictors for BCVA, after adjusting for all confounders and using multiple regressions analysis, we showed that PPV + tPA was a significant predictor for better visual outcome at all time points of FU and regardless of haemorrhage size, compared to PD + tPA. To the best of our knowledge this is the first time to report such analysis. SMH dimensions greater than 20 mm^2 were predictive of worse outcome only at 3 months post-surgery. Other studies also found that SMH dimension was a predictor for BCVA [15]. As for duration of SMH, in our study this factor was not a significant predictor for BCVA. Other studies report contrasting results with some studies that found a predictive value to SMH duration [16] and some studies that did not [17].

### Complications

When comparing complications between the two treatment modalities, we didn’t find a significant difference in the rate of cataract surgery within one year, re- bleeding, vitreous hemorrhage, or a second surgery. Implying to equal safety to both surgeries. This observation should be taken with the consideration that all surgeries in both centers were conducted by well experienced surgeons. However, these findings are not surprising since both treatment modalities has been reported to be safe with minimal rate of complications [13, 18].

### Study limitations

This was a real world data retrospective analysis. And as such, some of the baseline characteristics were significantly different among groups. To overcome this gap, multivariate analysis was conducted. Another discrepancy between groups in baseline characteristics was that for SMH larger than 50 mm^2, only one case was referred to PD while the rest were referred to PPV. To avoid a selection bias and to better clarify the analysis between two treatment groups, subgroup analysis was conducted only to SMH smaller than 20 mm^2 and those that were 20–50 mm^2. Thus, this study’s findings do not relate to SMH larger than 50 mm2.

## Conclusions

This study examined the surgical outcomes of PPV + tPA versus PD + tPA for SMH complicated by AMD. Referral to PPV + tPA exhibited a clear advantage with a trend of better BCVA at all time points as well as a significant predictor to better BCVA at all FU points for small and medium SMH. Moreover, BCVA gain was significantly higher in small SMH undergoing PPV + tPA.

While in large SMH the tendency to refer for PPV + tPA is higher than in small SMH in which the option of less invasive office-based procedure such as PD + tPA can be considered, the results of this study imply to a clear advantage for PPV + tPA in small and medium SMH and may be taken in consideration in management of small and medium SMH.

### Data availability statement

Raw data were generated at Sheba Hospital, Ophthalmology department, Tel Hashomer, Tel Aviv, and Tel Aviv medical center, Ophthalmology, Tel Aviv, IL. Derived data supporting the findings of this study are available from the corresponding author A.D, A.B on request.

## Data Availability

The authors in this study have access to the data that supports the findings of this study, such as patient information. The availability of these data, which were used under license for the current study, is restricted and not publicly available. The authors can provide additional raw data upon reasonable request, as it is not in violation of the given license.
